# How can we improve amniocentesis decision-making?

**DOI:** 10.1186/s13584-016-0060-0

**Published:** 2016-02-05

**Authors:** Lisa Soleymani Lehmann

**Affiliations:** National Center for Ethics in Health Care, Veterans Health Administration, 810 Vermont Avenue, NW Washington, DC, 20420 USA; Department of Medicine, Harvard Medical School, Boston, USA; Department of Global Health and Social Medicine, Harvard Medical School, Boston, USA; Department of Health Policy and Management, Harvard T.H. Chan School of Public Health, Boston, USA

## Abstract

The decision to have an amniocentesis entails a trade-off between a risk of procedure associated miscarriage and the benefit of obtaining diagnostic information to identify Down syndrome or other chromosomal aneuploidy. Ideally, this trade-off is informed by first and second trimester pre-natal screening tests, such that women with low risk screening test results are not encouraged to have an amniocentesis.

In a recent IJHPR article, Grinshpun-Cohen et al. surveyed 42 Israeli women without a medical indication for amniocentesis other than age. They found that one third of women who had a noninvasive serum screening test prior to amniocentesis did not even wait for the test results before electing to have the invasive procedure and 10 % of women did not have any serum screening test prior to amniocentesis.

There may be multiple reasons why women of advanced maternal age are not integrating screening risk information into their decision-making about amniocentesis. However, our understanding of those reasons is limited, as we don’t have information on the content of conversations between health care providers and women who are considering amniocentesis. We don’t know if health care providers counseled women on how screening risk information can inform their decision about whether or not to purse a diagnostic amniocentesis. Even if women with screening tests results suggestive of a low risk of Down syndrome were counseled not to pursue an amniocentesis, some women may have a preference for diagnostic information about a fetus’s Down syndrome status. Health care providers should, however, be encouraged to engage women in a process of shared decision making to ensure that women are informed and making deliberative decisions that meet their goals of care. Offering women a relatively new, cell-free fetal DNA test may provide reassurance that negates the impulse to have an amniocentesis. Public funding for amniocentesis for all women of advanced maternal age should continue as the decision to purse an amniocentesis is best determined by women who have to live with the consequences of their choice.

## Commentary

### Background

What factors should enter into a woman’s decision to undergo amniocentesis? Is the mere fact of advanced maternal age sufficient to justify the risk of miscarriage associated with the invasive diagnostic procedure? From a public policy perspective, should public funding cover amniocentesis for all women who are older than 37 years of age at the onset of their pregnancy, regardless of other risk factors? What should be the role of health care providers in helping women deliberate about the decision to pursue an amniocentesis?

In their important study on the effect of risk information on women’s decisions to undergo amniocentesis, which was recently published in the Israel Journal of Health Policy and Research, Grinshpun-Cohen et al. surveyed 42 women without a medical indication for amniocentesis other than age. They evaluated their knowledge and opinions regarding screening tests, risks of amniocentesis, and factors that affected the decision to pursue amniocentesis with an eye toward assessing if their decisions were informed. Surprisingly, one third of women who had a noninvasive serum screening test prior to amniocentesis did not even wait for the test results before electing to have the invasive procedure and 10 % of women did not have any serum screening test prior to amniocentesis. Furthermore, while most women could recall the risk of miscarriage associated with an amniocentesis, many could not recall their serum screening test results or their age-related Down syndrome risk. These findings suggest that women are not integrating objective risk estimates into their decision making about whether to pursue an amniocentesis.

These findings are consistent with a recent study of 3217 women in Greece which evaluated the impact of prenatal screening test results on the decision to undergo amniocentesis in women older than 35. Among women who had prenatal screening, 79 % were low risk for Down syndrome, but nevertheless decided to have an amniocentesis.

What might account for women’s disregard of objective risk information? Does it really imply that women are not being deliberative about the decision to have an amniocentesis? If objective risk estimates are not fueling women’s decisions to pursue an amniocentesis, what is? Risk communication is very complex and may be influenced by both health care providers’ and patients’ varying perceptions of risk information. The severity and consequences of the risk, as well as individual patient characteristics and framing of risk information influence decision-making [1, 2].

In the context of this study of women’s decision making about amniocentesis, we don’t know how health care providers counseled women about whether and how to integrate objective risk estimates into their decision making. It is unclear if women were given tailored options about screening for Down syndrome and if the implications of different screening approaches were explained. In a recent study by Srebnik et al., 44 % of Israeli clinicians recommended an amniocentesis on the basis of older age [3]. There are multiple approaches to screening and women’s preferences should influence the approach taken [4, 5].

Ultimately, it is women’s values that should drive the pre-natal screening process. Some women may value information about their fetus’s risk of Down syndrome during the first trimester even if it is associated with a higher procedure-related risk of miscarriage. For these women a screening test that combines maternal serum screening of beta-hCG and pregnancy-associated plasma protein-A (PAPP-A) with ultrasound measurement of fetal nuchal translucency will detect 85 % of Down syndrome fetuses at a 5 % false positive rate. Other women may have a stronger impulse to minimize the procedure-related pregnancy loss even if it means they won’t be able to identify a high-risk fetus during the first trimester. For such women, an integrated test that combines first trimester nuchal translucency, PAPP-A with second trimester quadruple serum markers will detect 90 % of Down syndrome fetuses and have a 1 % false positive rate. The down side of this approach is that the results are not available until the second trimester. Thus, women who are open to pregnancy termination and may have a preference for termination earlier in pregnancy will not be in a position to make a decision about termination until the second trimester. Still other women may place a strong value on being absolutely certain that they are not carrying an affected fetus. For such women, the results of a screening test won’t quell their fear and they may be reluctant to let a screening test that returns with a low risk of having an affected fetus dissuade them from pursing an invasive diagnostic procedure even if that procedure carries a risk of miscarriage.

It is generally recommended that women with screen negative results not pursue further diagnostic testing. For some women, however, even an excellent screening test will not be a substitute for a definitive diagnostic test. A negative screen only means that the risk of having a baby with Down syndrome is less than a specified cut off; it does not definitively dismiss the possibility of Down syndrome.

Did the one third of women in this study who did not await the results of their first and second trimester serum screening tests consciously decide to accept the trade off of possible procedure associated fetal loss for certainty that their fetus was not affected by a chromosomal abnormality? Were they counseled not to have an amniocentesis because of their low risk of having an affected fetus? We simply do not know the answer to these questions. Future research is needed to better understand the conversations that women have with their health care providers about pre-natal screening and amniocentesis. A study that captures the actual conversations that women are having with their clinicians about prenatal screening options, the implications of positive and negative screening tests and the role that individual women’s values play in decision making would provide a foundation for better understanding how women are currently being counseled and how we can improve the process of decision making surrounding amniocentesis.

Another option for women to consider is the relatively new screening test using a maternal plasma-based test for cell free fetal DNA during or after the 10^th^ week of pregnancy. Although it is not diagnostic, the high sensitivity and specificity of this DNA test in both high- and low-risk populations may reassure many women that their risk of carrying a Down syndrome fetus is so low that it allows them to avoid an amniocentesis [6]. Two studies support offering cell-free DNA screening to all pregnant women who desire screening [7, 8]. When compared with standard screening using ultrasound nuchal translucency and maternal serum markers, cell-free DNA screening of women with a mean gestational age of 12.5 weeks (range 10–14.3 weeks) had 100 % sensitivity for detection of Down syndrome and a false positive rate of 0.06 % [7].

Israel has a generous policy of funding amniocentesis for all women of who are 35 years of age or older. Although pregnant women may erroneously interpret this policy to imply that all women over 35 should have an amniocentesis, health care providers need tools to educate women about appropriate utilization of the test. Given the procedure associated risks of amniocentesis it is unlikely that informed women would opt for the procedure simply because it is funded by the government. Health care providers have an important role to play in helping women understand the complexity of prenatal screening and in guiding women through a process of shared decision making to arrive at a decision that is consistent with their personal goals and values. The policy of funding amniocentesis for all women of advanced maternal age is prudent in that many women of advanced maternal age may have a strong preference for the certainty of information associated with an amniocentesis. Continued government funding gives women and their health care providers the freedom to make high quality tailored clinical decisions without government interference.

A liberal public policy with regard to funding, however, comes with a significant responsibility for health care providers. As professionals, health care providers should be encouraged to uncover patients’ considered goals and educate patients about their prenatal screening options so that they can arrive at high quality deliberative medical decisions about amniocentesis. Women should be encouraged to undergo screening tests and incorporate these results into their decision making about a diagnostic amniocentesis. While it may be tempting to limit funding for amniocentesis to women who have a high-risk screen, such a policy is likely lead to worse outcomes for women and their children. It is women and their families who will have to care for a Down syndrome child and therefore it is their goals and values, not the government’s, which should drive clinical decision-making.

A study of patients facing surgical procedures found that informed patients opt for less surgery than those in “usual care” [9]. More informed patients seem to opt for less costly conservative treatment options. Instead of developing policies that restrict government funding, we should be creating decision aids to help women make high quality informed decisions and encouraging health care providers to engage patients in a process of shared decision making.

## Conclusions

Many women who undergo first or second trimester pre-natal screening are not using this information to inform their decision about amniocentesis. It is unclear if this is a consequence of a quest for certitude associated with a diagnostic procedure or if it is a failure of health care providers to engage women in shared decision making about pre-natal screening and amniocentesis. Future research should illuminate the content of conversations between women and health care providers to better assess the quality of decision-making regarding amniocentesis. While many women with low risk screening tests decide to purse an amniocentesis that carries a risk of miscarriage, restricting public funding of amniocentesis to women with advanced maternal age and high risk prenatal screening is unlikely to dissuade women who want definitive information on the status of their fetus from having an amniocentesis. Offering women cell-free fetal DNA testing may provide women with sufficient reassurance such that they don’t feel compelled to have an amniocentesis in the setting of low risk screening.
